# The ratio and interaction between neurotrophin and immune signaling during electroconvulsive therapy in late-life depression

**DOI:** 10.1016/j.bbih.2021.100389

**Published:** 2021-11-16

**Authors:** Dore Loef, Kristof Vansteelandt, Mardien L. Oudega, Philip van Eijndhoven, Angela Carlier, Eric van Exel, Didi Rhebergen, Pascal Sienaert, Mathieu Vandenbulcke, Filip Bouckaert, Annemiek Dols

**Affiliations:** a Amsterdam UMC, Location VUmc, Department of Psychiatry, Amsterdam Public Health Research Institute and Amsterdam Neuroscience, De Boelelaan 1117, 1118, 1081 HV, Amsterdam, the Netherlands; b GGZ InGeest Specialized Mental Health Care, Department of Old Age Psychiatry, Oldenaller 1, 1081 HJ, Amsterdam, the Netherlands; c University Psychiatric Center KU Leuven, Academic Center for ECT and Neuromodulation AcCENT, Leuvensesteenweg 517, 3070, Kortenberg, Belgium; d Donders Institute for Brain, Cognition and Behavior, Department of Psychiatry, Kapittelweg 29, 6525 EN, Nijmegen, the Netherlands; e Pro Persona Mental Health Institute, Department of Old Age Psychiatry, Nijmeegsebaan 61, 6525 DX, Nijmegen, the Netherlands; f Mental Health Care Institute GGZ Centraal, Westsingel 41, 3811 BB, Amersfoort, the Netherlands; g University Psychiatric Center KU Leuven, Department of Old Age Psychiatry, Leuven/Kortenberg, Belgium

**Keywords:** Electroconvulsive therapy (ECT), Depression, Brain-derived neurotrophic factor (BDNF), Interleukin-6 (IL-6), Tumor necrosis factor α (TNF-α)

## Abstract

**Background:**

Electroconvulsive therapy (ECT) is the most effective treatment for severe late-life depression (LLD), and several hypotheses on the precise working mechanism have been proposed. Preclinical evidence suggests that ECT induces changes in neurotrophin and inflammatory signaling and that these neurotrophic and inflammatory systems affect each other. We examine the relation, interaction, and ratio between the neurotrophic brain-derived neurotrophic factor (BDNF) and the proinflammatory cytokines interleukin-6 (IL-6) and tumor necrosis factor α (TNF-α), and depression severity during ECT.

**Methods:**

In this naturalistic longitudinal study, linear mixed models were used to analyze the relation between BDNF, IL-6, TNF-α, and depression severity (determined by the Montgomery-Åsberg Depression Rating Scale; MADRS) in 99 patients with severe LLD before ECT (T0), three weeks after the first ECT (T1), and one week after the last ECT (T2).

**Results:**

No significant association was found between BDNF, IL-6 and TNF-α, and MADRS scores at any time point. However, a significant interaction between TNF-α and BDNF in relation to MADRS was established (*p* ​= ​.020) at all time points. With higher levels of TNF-α, the relation between BDNF and MADRS becomes more negative. Furthermore, a higher ratio of TNF-α/BDNF was associated with a higher score on the MADRS (*p* ​= ​.007).

**Conclusion:**

A possible explanation for the absence of a significant coevolution between the proinflammatory cytokines and BDNF could be that the study design was unable to determine parameters shortly after ECT sessions. However, the TNF-α/BDNF ratio was positively associated with depression severity, and the association of BDNF-level and depression severity depended on the level of TNF-α. This suggests that the interaction and balance between neurotrophin and immune signaling, specifically BDNF and TNF-α, could be relevant in LLD. This could be a focus in future research regarding treatment and the working mechanism of ECT.

## Highlights

• IL-6 and TNF-α, BDNF, and depression severity were not significantly associated.
• The TNF-α/BDNF ratio is positively associated with depression severity.
• The association of BDNF-levels and depression severity depends on the level of TNF-α.
• Interplay between neurotrophin and immune signaling could be relevant in LLD.

## 1 Introduction

Electroconvulsive therapy (ECT) is the most effective treatment for severe late-life depression (LDD) (Kerner and Prudic, 2014). Several hypotheses on the working mechanism of ECT have been proposed (Bouckaert et al., 2014), each appointing different aspects essential for the therapeutic efficacy of ECT: generalized seizures, the normalization of neuroendocrine dysfunction, or increased hippocampal neurogenesis and synaptogenesis (Bolwig, 2011). Nevertheless, it is evident that neither complex psychiatric disorders nor the mechanism of action of their treatments can be explained by an imbalance in just a few chemical substances (Sienaert, 2014).

Major depression disorder (MDD) could be conceptualized as stress-elicited neuronal micro injury resulting in behavioral disturbances (Patas et al., 2014). As a result of the inflammatory response induced by this stress-related micro injury, microglia cells are activated and these in turn enable the secretion of cytokines such as interleukin-6 (IL-6) and tumor necrosis factor alpha (TNF-α) (Yrondi et al., 2018). The aim of this endogenous inflammation may be to induce neurotrophin release for tissue repair (Patas et al., 2014). This cascade of inflammatory response to depression related micro-injury inducing neurotrophin release may explain (natural) recovery of depression (Schulte-Herbrüggen et al., 2005). show that among an array of cytokines, only IL-6 and TNF-α specifically enhance brain-derived neurotrophic factor (BDNF) secretion in human monocytes. In another study, it was confirmed that TNF-α should be considered in mechanisms of BDNF-dependent neuronal plasticity (Bałkowiec-Iskra et al., 2011). Additionally, BDNF levels in patients with MDD with melancholic features were positively associated with IL-6 (Patas et al., 2014).

However, severe MDD could lead to a prolonged and detrimental state of inflammation. Hence, pathological inflammation is differentiated from advantageous inflammation by the intensity and timing of its appearance (van Buel et al., 2015). Recovery from MDD by ECT may be understood as ECT-induced controlled bouts of inflammation paving the way for neurorepair processes (van Buel et al., 2015). Neuroprotective properties of inflammation have been comprehensively described, and the significance of this role of inflammation in psychiatric disorders should be acknowledged (van Buel et al., 2015). Electroconvulsive shock (ECS) treatment, an animal model of ECT, generates transient and rapid microglia activation leading to a time-limited upregulation of proinflammatory cytokines (An and Shi, 2020). Since the upregulation is transient, the proinflammatory response results in neuroprotection and therapeutic effect rather than neuro-damage through cytokine toxicity and upregulated oxidative stress (An and Shi, 2020). Individual ECT sessions acutely and transiently upregulate inflammatory cytokines such as IL-6 and TNF-α and research results suggest that this stimulation results in an increase in neurotrophins such as BDNF (Guloksuz et al., 2014). According to the neurotrophin hypothesis of MDD, lower expression of BDNF causes reduced neurogenesis and synaptic plasticity (Brunoni et al., 2014). Subsequently, it is presumed that the increment in BDNF leads to neuro-repair processes and, thereby, clinical response. Aforementioned is a possible working mechanism of ECT in which bouts of inflammation may mobilize neuroprotection (Rush et al., 2016; van Buel et al., 2015). Therefore, the balance between inflammatory and neurotrophic cytokines may change during the course of ECT resulting in therapeutic effect, as a delicate balance exists between pro-inflammatory cytokines and neurotrophins and changes in this balance affect the brain (Johnson and Sharma, 2003).

Previously, we found that some degree of proinflammation (Carlier et al., 2019) and low serum levels of BDNF (van Zutphen et al., 2019) before ECT were associated with favorable outcome in the same cohort with severe LLD treated with ECT. Further research is needed to investigate to what extent the assumed mechanism of ECT in MDD applies to our specific LLD subgroup as the etiology of LLD is rather heterogeneous and immune changes emerge during aging (Dols et al., 2017; Gruver et al., 2007). Recently, research results showed that ECT response in LLD was not affected by age related brain changes such as hippocampal volume, white matter hyperintensities and amyloid accumulation (Bouckaert et al., 2019). In the present study, we hypothesize that over the course of ECT proinflammatory cytokine levels are positively associated with BDNF levels, and that this association is related to response (e.g. lower depression severity). Therefore, the relation between BDNF, IL-6, TNF-α, and depression severity before, during, and following ECT in severe LLD will be examined. Interaction and ratio between proinflammatory cytokines and BDNF are also taken into account, since better understanding of the interaction between proinflammatory cytokines and BDNF is needed and the proinflammatory cytokine/neurotrophin balance may play an important role in brain diseases (Johnson and Sharma, 2003; Lima Giacobbo et al., 2019).

## 2 Methods

### 2.1 Participants

In total, 110 older adults with a diagnosis of severe MDD according to the Diagnostic and Statistical Manual of Mental Disorders, Fourth Edition, Text Revision (DSM-IV-TR) criteria participated (Cooper, 1995). All patients were treated with ECT, primarily indicated on account of pharmacotherapy resistance, life-threatening symptoms, or excellent response to ECT during a previous depressive episode. Patients were recruited in two psychiatric hospitals: GGZ inGeest, Amsterdam, the Netherlands, and University Psychiatric Center, KU Leuven, Belgium. Exclusion criteria were a diagnosis of schizoaffective disorder, bipolar disorder or a major neurological illness such as Parkinson's disease, stroke and dementia. All disorders were diagnosed by a psychiatrist and established by the Mini International Neuropsychiatric Interview (Sheehan et al., 1998). Patients were assessed before ECT (T0), three weeks after the first ECT (T1), and one week after the last ECT (T2). The study protocol of Mood Disorders in Elderly treated with ECT (MODECT) was approved by both the Ethical Review Board of the VU University Medical Center and the Ethical Review Board of the Leuven University Hospitals, and was conducted according to the Declaration of Helsinki. Of the 110 individuals in MODECT, 99 patients with (partly) available data of interest (IL-6, TNF-α, BDNF levels) were eligible for the present study. These 99 individuals were included in all analyses executed. IL-6, TNF-α, and BDNF measurements were available from 97 patients at T0, 87 patients at T1 and 95 patients at T2. MADRS scores were available from 92 patients at T0, 81 patients at T1 and 97 patients at T2.

### 2.2 Clinical measurement

Demographic and clinical variables were obtained by means of a semi-structured interview. To determine the depressive symptom severity, the present study assessed the Montgomery Åsberg Depression Rating Scale (MADRS) on T0, T1, and T2 (Montgomery and Åsberg, 1979). The overall MADRS score ranges from 0 to 60 and a higher score represents a greater degree of depressive symptom severity.

### 2.3 ECT procedure

ECT was administered twice a week with a constant-current brief-pulse (0.5–1.0 ​ms) device: Thymatron System IV (Somatics, LLC, Lake Bluff, IL, USA). Concerning the administration of ECT, Dutch guidelines and standards were maintained (Van den Broek et al., 2010). Anesthesia was accomplished with intravenous administration of 0.2 ​mg/kg of etomidate and pursued by 1 ​mg/kg of succinylcholine. Treatments started with right unilateral ECT, and the stimulus intensity was determined by empirical dose titration at the first treatment. This implied six times the initial seizure threshold for right unilateral ECT and two and a half times seizure threshold for bilateral ECT. Motor and electroencephalographic (EEG) seizures were monitored, and when a motor seizure of fewer than 20 ​s occurred, it was considered inadequate, after which the dose was increased. Switching from unilateral to bilateral ECT took place when the clinical condition deteriorated or when there was no clinical improvement after six unilateral treatments according to the judgement of the treating psychiatrist. Response to treatment was defined as a 50.0% or more reduction in total MADRS score. ECT was stopped upon achieving remission which was set by a score of less than 10 on the MADRS at two sequent measurements with a week interval. ECT was also stopped when patients displayed no further progress in clinical condition during the last two weeks of ECT sessions after a minimum of six unilateral and six bilateral treatments. Psychotropic drugs were tapered off at least one week before the start of ECT (n ​= ​60) or, if necessary, kept stable from six weeks before the start of ECT (n ​= ​39).

### 2.4 Sampling and cytokine measurements

Blood samples were taken at the three time points mentioned above (T0, T1, T2) to determine IL-6, TNF-α and BDNF cytokine levels. Between 0730 and 0930 ​h, 50 ​mL of blood was collected and withdrawn in vacuum tubes after an overnight fast. Serum was instantly separated and stored at −85 ​°C until being assayed in seven to 43 months after collection. Using the Single Molecule Array (Simoa) technology and the Simoa HD-1 Analyzer, the IL-6 and TNF-α serum levels were determined with a three-step multiplex digital immunoassay, the Simoa Human Cytokine Three-Plex A assay kit (Quanterix; cat.#101160; MA, USA). For IL-6, the intra-assay reproducibility was 4.3%, and the inter-assay reproducibility, for which the coefficient of variation was based on data from ten runs, was 5.9%. For TNF-α, this was 4.2% and 5.3%, respectively. Upon thawing, the samples were mixed by inverting, spun at 4 ​°C (10’; 10000*g*), and assayed instantly. Serum samples containing either low or high cytokine levels were included in each plate run to control quality. BDNF levels were also determined by using the separated serum from blood samples. To accurately increase the detectable BDNF in a dilution-dependent way undiluted serum was acid treated. Greiner Bio-One high affinity 96-well plates were used. Serum samples were diluted 100 times, and the resulting absorbance was read in duplicate using a Bio-Rad (Hercules, CA, USA) Benchmark microplate reader at 450 ​nm. For IL-6, the lower limit of detection was 0.006 ​pg/mL and the lower limit of quantification was 0.011 ​pg/mL. For TNF-α, this was 0.011 ​pg/mL and 0.051 ​pg/mL, respectively. No patient had BDNF values that were below the reliable detection threshold of 1.56 ​ng/mL. The serum levels were determined in the University Medical Center of Maastricht using the Emax Immuno Assay System from Promega according to the manufacturer's protocol (Madison, WI, USA).

### 2.5 Statistical analysis

Data were analyzed using the software “Statistical Package for the Social Sciences” (IBM SPSS Statistics for Windows, version 25.0, 2017). IL-6 values ranged from 0.0 to 145.9 ​pg/mL (*M* ​= ​5.43, *SD* ​= ​10.9), and the data within this variable contained three outliers (61.8, 66.2, and 145.9). These three outliers were not included in the analyses, since these values were highly deviant (more than five to 14 times the standard deviation from the mean) and a result that is merely based on these outliers is less valuable. Moreover, to avoid bias as a result of inflammatory disease and acute infection or treatment such extreme outliers were excluded. Residuals of all analyses were checked to be normally distributed. TNF-α values ranged from 0.0 to 19.7 ​pg/mL (*M* ​= ​4.51, *SD* ​= ​2.11), and BDNF values ranged from 4.03 to 35.6 ​ng/mL (*M* ​= ​18.3, *SD* ​= ​6.65). To investigate the relation between IL-6, TNF-α, BDNF, and MADRS, multilevel or linear mixed models (LMM) were estimated with repeated measurements (level 1) being nested within patients (level 2). LMMs were chosen because they: i) use all available data, ii) can properly account for correlation between repeated measurement on the same subject; iii) allow multivariable analysis; and iv) can handle missing data adequately (Gueorguieva and Krystal, 2004). The relation between the proinflammatory cytokines and BDNF was analyzed using LMM with BDNF as a dependent variable and IL-6 and TNF-α as an independent variable. The relation of proinflammatory cytokines and BDNF with MADRS was analyzed using LMM with MADRS as a dependent variable and BDNF and consecutively IL-6 and TNF-α as independent variables. Interactions between proinflammatory cytokines and BDNF were taken into account. Subsequently, the balance between the proinflammatory cytokines and BDNF over time were analyzed using LMM with the ratios as dependent variables and the time as independent variables. Finally, the relation between the ratio of proinflammatory cytokine/BDNF and MADRS was analyzed using LMM with MADRS as a dependent variable and consecutively the ratio IL-6/BDNF and ratio TNF-α/BDNF as an independent variable. All analyses were adjusted for site (Amsterdam or Leuven), age, time points, medication use during ECT (yes or no), switching from unilateral to bilateral electrode placement (yes or no), and total number of received ECT sessions. Analyses where MADRS was not the dependent variable were also adjusted for remission from MDD after the ECT course. These variables were included in the model instead of performing stratification in order to prevent a dramatic increase in the number of subgroups which would have highly unbalanced sample sizes. After consideration, BMI and smoking status were not included as adjusted factors, as no relation with ECT response have been reported. Furthermore, no significant associations were found between smoking status or BMI and the independent and dependent variables (BDNF, IL-6, TNF-α, MADRS) neither at baseline nor with repeated measurements. Interactions between remission and dependent variables were taken into account. If an interaction term was not significant (*p* ​≥ ​.050), it was removed from the model to interpret the main effect. Model selection was based on likelihood ratio tests and information criteria.

## 3 Results

### 3.1 Clinical characteristics and covariates

Clinical characteristic are shown in Table 1. The age of the included 99 patients ranged from 55 to 92 (*M* ​= ​72.8, *SD* ​= ​8.29) with 17 patients aged 55–64, 36 patients aged 65–74, 40 patients aged 75–84, and six patients aged 85–94. 66.7% of patients were female and the mean administered number of ECT sessions per patient was 12 (*SD* ​= ​5.53). During treatment course, 31.3% of patients switched to bilateral ECT due to inadequate response with right unilateral ECT. Response was seen in 83.9% of patients and 71.1% achieved remission. The 99 patients in our study sample were included in all linear mixed model analyses executed. The following covariates were significant in analyses where MADRS was the dependent variable: age, time points, switching from unilateral to bilateral electrode placement and total number of received ECT sessions. MADRS scores decreased significantly over time and a lower MADRS score was associated with a higher age. Also, at T1 and T2 a higher MADRS score was associated with a higher total number of received ECT sessions. Patients switching from unilateral to bilateral electrode placement had a significantly higher MADRS score at T1 and T2. The covariate site was significant in the analyses with BDNF and ratio IL-6/BDNF as dependent variables. Patients from Amsterdam had a significantly higher BDNF-levels, F(1, 97.89) ​= ​38.6, *p* ​< ​.001, and lower ratios IL-6/BDNF, F(1, 95.24) ​= ​5.07, *p* ​= ​.027, than patients from Leuven. A higher age was also associated with a higher ratio IL-6/BDNF, F(1, 97.87) ​= ​7.50, *p* ​= ​.007. All covariates not mentioned were not significantly associated with the dependent variable in other analyses. Remission from MDD (which was added as covariate in analyses where MADRS was not the dependent variable) and medication use were not significantly associated with dependent variables.

**Table 1:** Demographics and clinical characteristics of the study sample.

	Study sample (*N* ​= ​99)
Age, mean (SD)	72.8 (8.32)
Female, *n* (%)	66 (66.7)
Amsterdam, *n* (%)	56 (56.6)
Age at onset of first depressive episode, late (>55 years), *n* (%)	56 (56.6)
Duration of current episode in months, median (IQR)	6.00 (10)
Number of previous antidepressants failures in current episode, mean (SD)	1.93 (1.14)
Highest resistance score of previous antidepressant treatments measured by the ATHF, mean (SD)	3.12 (1.34)
Medication use ceased, *n* (%)	60 (60.6)
Switched to bilateral ECT, *n* (%)	31 (31.3)
Total ECT sessions, mean (SD)	11.8 (5.53)
MADRS score at T0 (available n ​= ​92), mean (SD)	33.6 (9.14)
MADRS score at T1 (available n ​= ​81), mean (SD)	18.3 (10.5)
MADRS score at T2 (available n ​= ​97), mean (SD)	9.64 (9.27)

Note: ATHF ​= ​Antidepressant Treatment History Form (Sackeim, 2001), ECT ​= ​electroconvulsive therapy; MADRS ​= ​Montgomery Åsberg Depression Rating Scale (Montgomery and Åsberg, 1979); T0 ​= ​before ECT; T1 ​= ​three weeks after the first ECT; T2 ​= ​one week after the last ECT.

### 3.2 Coevolution of IL-6, TNF-α, BDNF, and depression severity

**Fig. 1:**
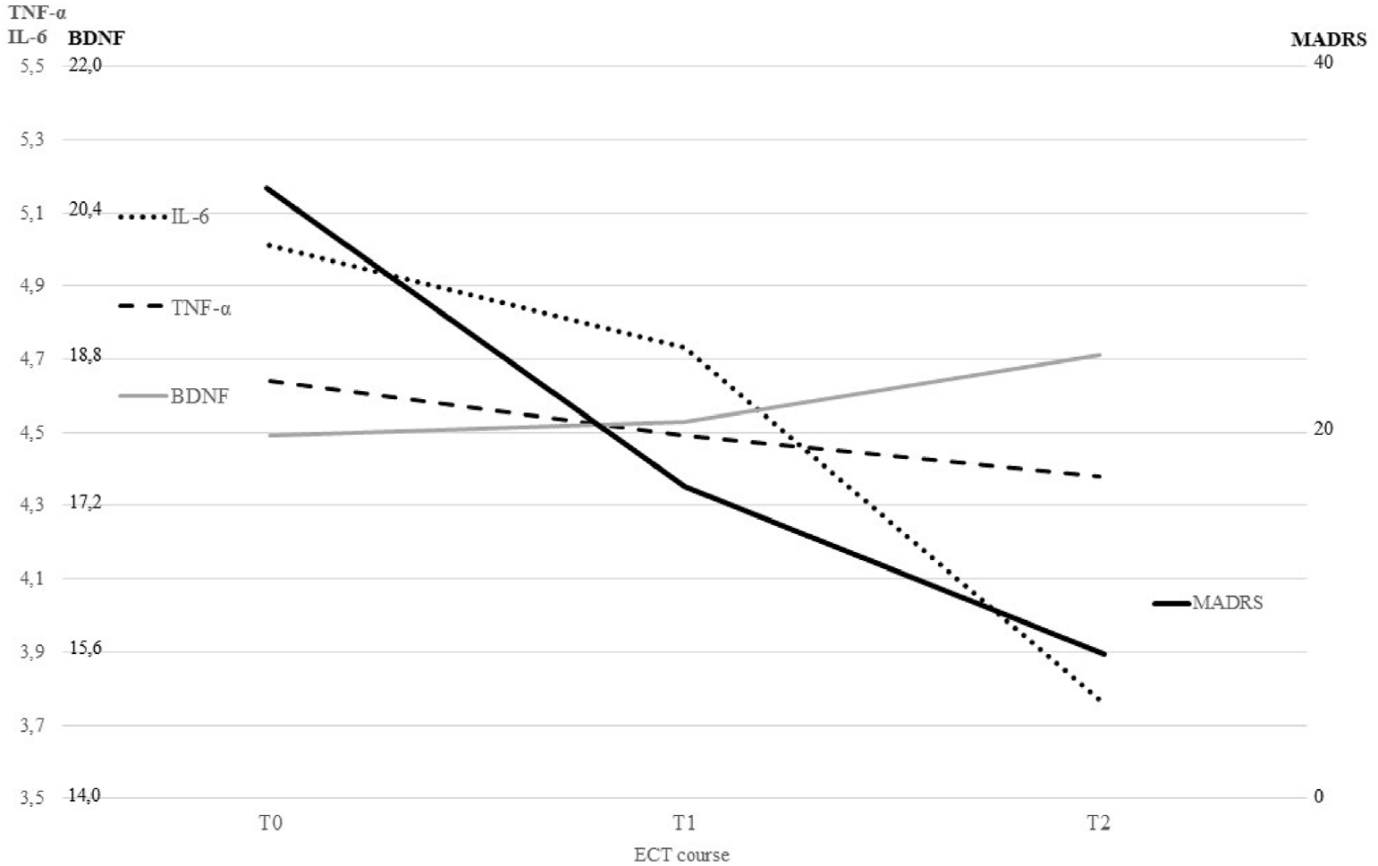
Alterations in interleukin-6 (IL-6), tumor necrosis factor alpha (TNF-α), brain-derived neurotrophic factor (BDNF) and the Montgomery Åsberg Depression Rating Scale (MADRS) score over the course of electroconvulsive therapy (ECT): before ECT (T0), three weeks after the first ECT (T1), one week after the last ECT (T2).

The evolution of IL-6, TNF-α, BDNF, and MADRS over the course of ECT is shown in Fig. 1. To examine the relation between IL-6, TNF-α, BDNF, and MADRS over the course of ECT three LMMs were executed. The first LMM showed no significant association between IL-6 and BDNF, F(1, 260.45) ​= ​1.83, *p* ​= ​.177, over the course of ECT, nor between TNF-α and BDNF, F(1, 271.67) ​= ​1.04, *p* ​= ​.309, and these associations were not significantly different in remitters than in non-remitters. The next LMMs disclosed no significant association between IL-6 and MADRS, F(1, 212.10) ​= ​0.95, *p* ​= ​.331, BDNF and MADRS, F(1, 206.77) ​= ​0.26, *p* ​= ​.611, and TNF-α and MADRS, F(1, 215.59) ​= ​2.35, *p* ​= ​.127. No interaction between IL-6 and BDNF was found, but the interaction between TNF-α and BDNF was significant, F(1, 179.15) ​= ​5.55, *p* ​= ​.020. Thus, the effect of BDNF on MADRS depends on the level of TNF-α. With higher levels of TNF-α the relation between BDNF and MADRS becomes more negative (lower BDNF levels with higher MADRS scores), while with lower levels of TNF-α the relation between BDNF and MADRS disappears.

### 3.3 Evolution of ratio proinflammatory cytokine/BDNF over time and association with depression severity

**Fig. 2:**
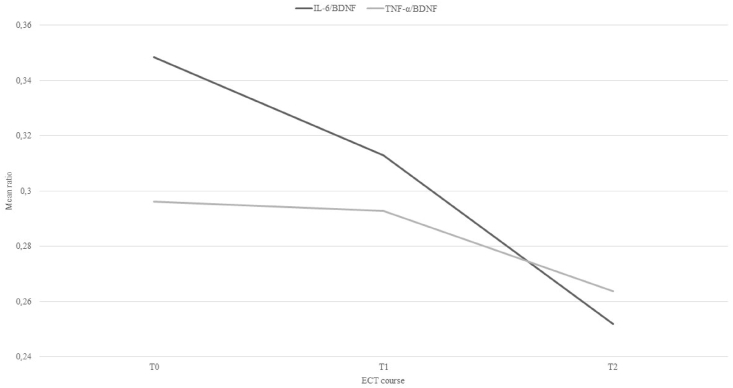
Alterations in the ratios interleukin-6/brain-derived neurotrophic factor (IL-6/BDNF) and tumor necrosis factor alpha/brain-derived neurotrophic factor (TNF-α/BDNF) over the course of electroconvulsive therapy (ECT): before ECT (T0), three weeks after the first ECT (T1), one week after the last ECT (T2).

The LMM for the evolution over the different time points of both the ratio IL-6/BDNF and the ratio TNF-α/BDNF disclosed no significant differences, F(2, 93.75) ​= ​2.65, *p* ​= ​.076 and F(2, 166.20) ​= ​1.35, *p* ​= ​.261, respectively (Fig. 2). Moreover, no interaction between remission and the ratios was observed. Thus, the course of the ratios over time was not significantly different in remitters than in non-remitters. The proinflammatory cytokine/neurotrophin balance in relation with depression severity over the course of ECT was examined. No significant association between the ratio IL-6/BDNF and MADRS was found, F(1193.61) ​= ​2.12, p ​= ​.147. However, a significant association was established between the ratio TNF-α/BDNF and MADRS, F(1, 210.43) ​= ​7.45, p ​= ​.007. A higher ratio of TNF-α/BDNF was associated with a higher score on the MADRS.

## 4 Discussion

In the present study IL-6, TNF-α, BDNF, and depression severity before, during, and after ECT were examined in LLD. No significant association between IL-6, TNF-α, BDNF, and depression severity was established before, during and after ECT. A significant association between the ratio of TNF-α/BDNF and depression severity was found: the higher the TNF-α/BDNF ratio, the greater the depression severity. Interestingly, no significant change in the proinflammatory cytokine/neurotrophin ratios over the course of ECT was found despite the significant decline of depression severity over time. However, a significant interaction between TNF-α and BDNF was demonstrated: the association of BDNF-level and depression severity depends on the TNF-α level.

### 4.1 Association of proinflammatory cytokines and BDNF

No significant coevolution was found between the proinflammatory cytokines and BDNF. This finding contradicts research results that show a positive association between the proinflammatory cytokines and BDNF in various study samples (Bałkowiec-Iskra et al., 2011; Patas et al., 2014; Schulte-Herbrüggen et al., 2005), possibly as a consequence of the dissimilarity in study samples and sampling time points in the study protocol. The study of (Schulte-Herbrüggen et al., 2005) demonstrated that BDNF secretion in human monocytes can be specifically enhanced by IL-6 and TNF-α. However, this process is observed in cells isolated from healthy individuals. Additionally, the finding that TNF-α increases BDNF expression was affirmed in rat neurons (Bałkowiec-Iskra et al., 2011). (Patas et al., 2014) showed that BDNF levels are positively associated with IL-6 levels in melancholic MDD patients with a mean age of 41.9. At the same time, research results suggest that IL-6 levels are negatively associated with BDNF levels in cancer patients with depression (Jehn et al., 2015). This discrepancy could indicate a complicated versatile relation between proinflammatory cytokines and BDNF. Furthermore, it could be possible that the relation between cytokines and BDNF is not manifest in this specific subgroup with LLD since the BDNF system could become less responsive with age (Bouckaert et al., 2016). This is underlined by the findings that BDNF levels in plasma of 140 adults significantly decreased with increasing age and that the BDNF system was affected in aged rats which included reduced transcription and impaired protein synthesis (Calabrese et al., 2013; Lommatzsch et al., 2005). Moreover, immune changes during aging, known as immunosenescence, could be an underlying phenomenon resulting in different immune responses in this specific subgroup as for example aged microglia have a decreased ability to enable the secretion of TNF-α and IL-6 (Gruver et al., 2007). Importantly, the design of the present study was inadequate to determine cytokines and BDNF levels shortly after ECT sessions, while research results suggest that after a single ECT session cytokine levels are increased over the following 3-h time point and then return to baseline in 24 ​h (Guloksuz et al., 2014). Perhaps, a relation between cytokines and BDNF exists during these controlled bouts of inflammation induced by ECT, that the present study was not able to measure. Therefore, it is advisable that future research examines the relation between cytokines and BDNF shortly after administration of ECT.

### 4.2 Coevolution of cytokines, BDNF, and depression severity

A recent meta-analysis found that during ECT treatment there is an initial pro-inflammatory response with increased IL-6 levels eventually followed by decreased IL-6 and TNF-α levels. A possible interpretation given by Gay et al. is that increased IL-6 levels in response to ECT induce neuroplastic change and the eventual decrease in IL-6 and TNF-α levels could be related to recovery of depression or to an anti-inflammatory response of ECT. However, in the present study no significant coevolution was found between IL-6, TNF-α, BDNF, and depression severity. Previously, data from the MODECT study indicated that LLD patients with lower levels of BDNF are more likely to achieve remission with ECT (van Zutphen et al., 2019). Nonetheless (Bouckaert et al., 2016), already demonstrated the lack of correlation between BDNF levels and depression severity over the course of ECT. A possible explanation may be that the relation between BDNF and the pathophysiology of MDD is much more complex than described and involves other neuroendocrine mechanisms and genetic factors (Rapinesi et al., 2015). However, we found a significant interaction between TNF-α and BDNF levels in relation to depression severity, suggesting that the effect of BDNF levels on depression severity depends on the level of TNF-α. Thus, future research should consider the interaction between BDNF and TNF-α in relation to LLD.

### 4.3 Proinflammatory cytokine/BDNF balance

Although no significant association is found between the ratio IL-6/BDNF and depression severity, a significant association between the TNF-α/BDNF and depression severity is established. The latter suggests that the proinflammatory cytokine/neurotrophin balance plays an important role in brain diseases (Johnson and Sharma, 2003). The present study demonstrates that the higher the ratio TNF-α/BDNF, the greater the severity of depression. Because of the significant association between ratio TNF-α/BDNF and depression severity together with the significant decrease in depression severity during the ECT-course in our cohort (Bouckaert et al., 2016), one would expect a decrease in the ratio TNF-α/BDNF as well. Although the ratio TNF-α/BDNF decreased over the course of ECT, this decrease was not significant and moreover the evolution of the ratio was not significantly different for remitters than for non-remitters. However, due to the high remission rates in the present study sample only 28 patients were classified as non-remitters resulting in less power to discriminate between remission groups. Furthermore, a possible explanation could be that in the present study sample with a mean decrease of 24.1 points in MADRS score after ECT, the decline in depression severity is strong to an extent that only a relatively marginal reduction in the ratio TNF-α/BDNF is needed. So if a small change in this delicate proinflammatory cytokine/neurotrophin balance would have a substantial effect on depression severity, it could explain why no significant decline in the ratio TNF-α/BDNF is observed. The underlying theory may be that the working mechanism of ECT induces a subtle decrease in the ratio TNF-α/BDNF and this in turn contributes to the therapeutic effect in LLD.

To our knowledge this is the first study that used the proinflammatory cytokine/BDNF ratios in relation to depression severity during and following ECT. The balance between immune and neurotrophin signaling may be relevant in LLD. The present study was designed to examine the relation between two proinflammatory cytokines, BDNF, and depression severity, and despite the lack of association, the interaction and balance between TNF-α and BDNF in relation with depression severity could underlie a broader psycho-neuroendocrine mechanism of action.

### 4.4 Limitations

The results of the present study should be interpreted in the light of its limitations. On the basis of the present findings, it may not be possible to demonstrate a relation between cytokine and BDNF levels induced by ECT because the study design was incompetent to determine cytokine and BDNF levels shortly after ECT sessions. Additionally, the present study examined LLD and future research should include MDD patients of all ages. Furthermore, differentiating between remitters and non-remitters was more difficult in the present study as relatively few patients did not remit and even less did not respond to ECT at all. Despite its limitations, with a sample of 99 patients the present study is fairly large for an ECT study. Furthermore, a strength of the present study is the careful evaluation and diagnosis of patients by a psychiatrist with establishment by the Mini International Neuropsychiatric Interview. Due to the exclusion of patients with bipolar or schizoaffective disorder the effect of ECT on unipolar depression could be more clearly considered.

## 5 Conclusions

The theory that ECT promotes a targeted elevation of the proinflammatory cytokine levels and thereby induce a neurogenic process via BDNF leading to clinical response in LLD could not be supported, since no significant coevolution between the proinflammatory cytokines, BDNF, and depression severity was found. However, sampling closer to ECT sessions is advised to study the more acute effect of ECT and is recommended for further studies to increase our understanding of the neurobiology of ECT.

Nevertheless, our findings that the TNF-α/BDNF ratio was positively associated with depression severity, and that the association of BDNF-levels and depression severity depended on the level of TNF-α suggest that the interaction and balance between neurotrophin and immune signaling, specifically BDNF and TNF-α, could be relevant in LLD. This could be a focus in future research regarding LLD treatment and the mechanism of action of ECT.

## Declaration of competing interest

The authors declare that they have no known competing financial interests or personal relationships that could have appeared to influence the work reported in this paper.

## Funding sources

This research did not receive any specific grant from funding agencies in the public, commercial, or not-for-profit sectors.
